# The Rise of Toroidal Electrodynamics and Spectroscopy

**DOI:** 10.1021/acsphotonics.2c01953

**Published:** 2023-03-15

**Authors:** Nikolay I. Zheludev, David Wilkowski

**Affiliations:** † Optoelectronics Research Centre, University of Southampton, Southampton, SO17 1BJ, United Kingdom; ‡ Centre for Disruptive Photonic Technologies, SPMS, The Photonics Institute, Nanyang Technological University, Singapore 637371, Singapore; § Centre for Quantum Technologies, National University of Singapore, Singapore 117543, Singapore; ∥ MajuLab, International Joint Research Unit, UMI 3654, CNRS, Université Côte d’Azur, Sorbonne Université, National University of Singapore, Nanyang Technological University, Singapore 639798, Singapore; ⊥ Hagler Institute for Advanced Studies, Texas A&M University, College Station, Texas 77843, United States

## Abstract

Toroidal electrodynamics is now massively influencing research in toroidal (Marinov et al. *New J. Phys.***2007**, *9*, 234; Basharin et al. *Phys. Rev. X***2015**, *5*, 011036; Jeong et al. *ACS Photonics***2020**, *7*, 1699) and anapole metamaterials (Basharin et al. *Phys. Rev. B***2017**, *95*, 035104; Wu et al. *ACS Nano***2018**, *12*, 1920), optical properties of nanoparticles (Miroshnichenko et al. *Nature Commun.***2015**, *6*, 8069; Gurvitz et al. *Laser Photonics Rev.***2019**, *13*, 1800266), plasmonics (Ogut et al. *Nano Lett.***2012**, *12*, 5239; Yezekyan et al. *Nano Lett.***2022**, *22*, 6098), sensors (Gupta et al. *Appl. Phys. Lett.***2017,***110*, 121108; Ahmadivand et al. *Mater. Today***2020**, *32*, 108; Wang et al. *Nanophotonics***2021**, *10*, 1295; Yao et al. *Photonix***2022**, *3*, 23), and lasers (Huang et al. *Sci. Rep.***2013**, *3*, 1237; Hwang et al. *Nanophotonics***2021**, *10*, 3599), while a recent publication on toroidal optical transitions in hydrogen-like atoms (Kuprov et al. *Sci. Adv.***2022**, *8*, eabq7651) promises to launch a new chapter in spectroscopy. In this Viewpoint, we review these progresses

## Electric, Magnetic, and Toroidal Multipole Expansion

**Figure 1:**
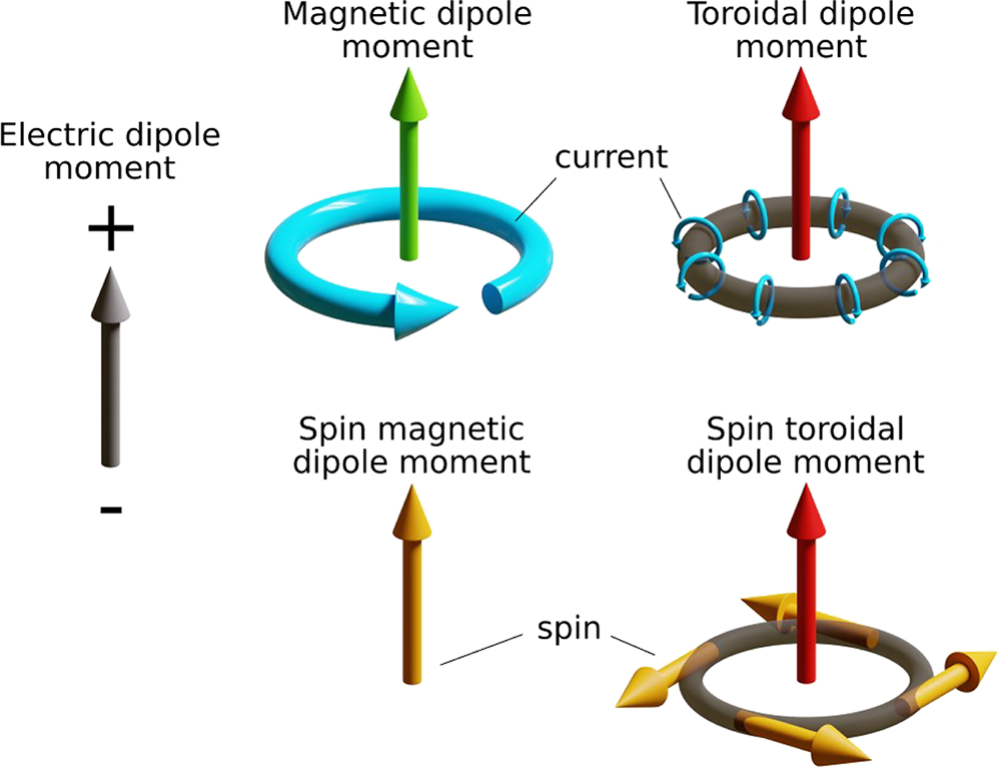
Schematic illustrations of electric, magnetic, and toroidal dipoles in classical electrodynamics. In relativistic physics, apart from magnetic and toroidal moments induced by charge currents, spin must be considered because it can also contribute to the toroidal dipole moment. Figure was extracted with permission from ref (16). Copyright 2022 AAAS.

The interactions of electromagnetic radiation with matter underpin some of the most important technologies today: from telecommunications to information processing and data storage; from spectroscopy and imaging to light-assisted manufacturing. Our understanding and description of the electromagnetic properties of matter traditionally involve the concept of electric and magnetic dipoles, as well as their more complex combinations, known as multipoles. Within this framework, termed the multipole expansion, electromagnetic media can be represented by a set of point-like multipole sources, commonly the families of the electric and magnetic moments, which can be represented by oscillating charges and loop currents, respectively. Dynamic toroidal multipoles constitute a third independent family of elementary electromagnetic sources, rather than an alternative multipole expansion or higher-order corrections to the conventional electric and magnetic multipoles.^17^ Classical toroidal dipole consists of current loops lying on a torus as shown in Figure 1. Combinations of torus loop currents lead to toroidal multipole terms. We note that toroidal multipoles are not a source of electric and magnetic fields if loop currents are time-independent, but radiates electromagnetic field when currents vary in time.

## Toroidal Resonances, Anapoles, and Supertoroidal Pulses

Observations of dynamic toroidal excitations are complicated by the presence of the electric and magnetic multipoles in the material’s response. The first observation of a toroidal dipole absorption resonance was reported in 2010 in a metamaterial, an artificial electromagnetic medium structured on the subwavelength scale.^18^ The electric and magnetic fields radiation pattern of the toroidal dipole in the far-field is identical to that of an electric dipole, although the corresponding charge-current configurations are different. Hence, a coherent superposition of dynamic electric and toroidal dipoles can be realized in a way that the radiated fields by the two dipoles interfere destructively. Nonradiating configurations of this type are now known as dynamic anapoles and were first observed in 2013 using a microwave metamaterial.^19^ They have shown that the destructive interference between coherently oscillating electric and toroidal dipoles provides a new mechanism of electromagnetic transparency, yielding narrow transmission lines.

Excitations of toroidal topology can also exist as electromagnetic field propagation with speed of light in free space. They are known as Toroidal Light Pulses or “light donuts”. They are nontransverse types of electromagnetic pulse that are radically different from conventional transverse electromagnetic waves. In a way, the Toroidal Light Pulses are propagating counterparts of localized toroidal dipole excitations in matter. Such pulses were recently generated by converting transverse electromagnetic pulses into “light donuts” through the interaction with tailored nanostructured meta-surfaces.^20^ The toroidal light pulses, their space–time coupling and their light–matter interactions involving anapoles, localized space–time coupled excitations, and toroidal qubits are of growing interest for the fundamental science of light and applications. Moreover, an extended family of electromagnetic excitation, the supertoroidal electromagnetic pulses has been introduced, in which the “light donut” pulse is just the simplest member. The supertoroidal pulses exhibit a skyrmionic structure of the electromagnetic fields, multiple singularities in the Poynting vector maps and fractal-like distributions of energy backflow.^21^

## Direct Toroidal Excitations in Atomic Physics

A recent work from Ilya Kuprov, David Wilkowski, and Nikolay Zheludev predicts that toroidal excitations are present also in the atom–light interaction,^16^ going against the common belief that atomic emission or absorption spectra are solely generated by electric and magnetic multipoles expansions.

Strict conservation laws govern the electromagnetic interaction with atoms through the selection rules of optical transitions. They indicate how two specific levels, among the atomic energy spectrum, can be coupled, through a single-photon transition. It turns out that toroidal dipole transitions have different selection rules than its electric and magnetic counterparts. Using Einstein’s special relativity in light–atom interaction, it was shown that the spin contribution to the toroidal dipole (see Figure 1) opens up new toroidal excitation channels in atoms. The account of the spin in the interaction of light with atoms yields the selection rules that makes transition between certain atomic levels easier to isolate from the background distinguishing them from more regular electromagnetic transitions.

The ultimate challenge is to find an atom with a proper set of two energy levels for a direct observation of toroidal transition where possible stronger electric and magnetic coupling are suppressed by selection rules. It appears that alkali atoms immersed into a strong static magnetic field can offer such energy levels. The role of the magnetic field is to decoupled the spin and orbital momenta of the atom, to address the relativistic toroidal term. This decoupling is facilitated for light atoms like Hydrogen and Lithium. An experimental implementation on Lithium’s Rydberg states is currently being developed at the Centre for Disruptive Photonic Technology of Nanyang Technological University in Singapore. In our view, future successful experiments in this field will open a new chapter in spectroscopy.

## Challenges and Opportunities

We believe that the future challenges and exciting opportunities for toroidal electrodynamics and spectroscopy are (1) in detecting high-order toroidal multipoles that shall be easier to achieve in structured media and metamaterials with lattice parameter close to the wavelength of light; (2) in developing toroidal spectroscopies of molecular and macromolecular systems possessing elements of toroidal symmetry; (3) in investigating yet unsettled question of reciprocity of interactions of toroidal excitations of different orders^22^ and electromagnetic forces involving toroidal excitations; (4) in developing spectroscopies of transient space-time nonrepairable excitations in matter induced by toroidal and supertoroidal pulses of light; (5) in designing new schemes for conversion of conventional laser pulses into toroidal and supertoroidal light pulses and lasers that directly generating such pulses; (6) in investigating the extended family of anapoles involving higher order toroidal excitations; (7) in searching for practical electromagnetic energy storage solutions based on anapoles and in exploring anapole qubits; (8) in developing quantum optics of space-time nonseparable toroidal pulses; (9) in searching toroidal emission signature in astrophysical electromagnetic signal.

## Data Availability

All data needed to evaluate the conclusions in the paper are present in the paper.
